# Insomnia - treatment pathways, costs and quality of life

**DOI:** 10.1186/1478-7547-9-10

**Published:** 2011-06-21

**Authors:** Guy W Scott, Helen M Scott, Karyn M O'Keeffe, Philippa H Gander

**Affiliations:** 1 School of Economics and Finance, Massey University, Wellington, New Zealand; 2 ScottEconomics, Wellington, New Zealand; 3 Sleep/Wake Research Centre, Massey University, Wellington, New Zealand

## Abstract

**Background:**

Insomnia is perhaps the most common sleep disorder in the general population, and is characterised by a range of complaints around difficulties in initiating and maintaining sleep, together with impaired waking function. There is little quantitative information on treatment pathways, costs and outcomes. The aims of this New Zealand study were to determine from which healthcare practitioners patients with insomnia sought treatment, treatment pathways followed, the net costs of treatment and the quality of life improvements obtained.

**Methods:**

The study was retrospective and prevalence based, and was both cost effectiveness (CEA) and a cost utility (CUA) analysis. Micro costing techniques were used and a societal analytic perspective was adopted. A deterministic decision tree model was used to estimate base case values, and a stochastic version, with Monte Carlo simulation, was used to perform sensitivity analysis. A probability and cost were attached to each event which enabled the costs for the treatment pathways and average treatment cost to be calculated. The inputs to the model were prevalence, event probabilities, resource utilisations, and unit costs. Direct costs and QALYs gained were evaluated.

**Results:**

The total net benefit of treating a person with insomnia was $482 (the total base case cost of $145 less health costs avoided of $628). When these results were applied to the total at-risk population in New Zealand additional treatment costs incurred were $6.6 million, costs avoided $28.4 million and net benefits were $21.8 million. The incremental net benefit when insomnia was "successfully" treated was $3,072 per QALY gained.

**Conclusions:**

The study has brought to light a number of problems relating to the treatment of insomnia in New Zealand. There is both inadequate access to publicly funded treatment and insufficient publicly available information from which a consumer is able to make an informed decision on the treatment and provider options. This study suggests that successful treatment of insomnia leads to direct cost savings and improved quality of life.

## Background

Insomnia is a disorder defined by difficulty initiating or maintaining sleep, or non-restorative sleep, along with impaired daytime function. These problems arise despite adequate time and opportunity for sleep [1,2]. Insomnia may occur as primary insomnia or insomnia comorbid to other medical or psychological conditions, substance abuse, or other sleep disorders. The outcomes of untreated insomnia are not well understood but it is known that insomnia is associated with a number of adverse health outcomes such as poor physical health, poor mental health including symptoms of anxiety and depression, and decreased quality of life [3,4]. There is currently no systematic national approach to insomnia diagnosis or treatment in New Zealand, and no requirement for treatment providers to have formal training or registration.

### Aims

The study aims are encapsulated in the following questions.

Policy question: in New Zealand, from which healthcare practitioners do patients seek treatment for insomnia, to whom are they referred, and what is the net cost and provider-assessed outcome of this treatment?

Research question: What are the effects of successful insomnia treatment on quality of life and health resource utilisation?

### Literature review

The following search engines were used to identify the literature that investigated the economic dimensions of insomnia; MEDLINE, Cochrane Library, AHRQ (Agency for Healthcare Research and Quality) Google Scholar and relevant New Zealand and Australian sites. Key words searched on included the following used alone and in various combinations; insomnia, cost, economic, analysis, Australia, New Zealand, UK, USA, America.

We reviewed and summarised the main findings of relevant papers published from 1996 onwards. The literature was then grouped into four categories; those papers that considered the prevalence of insomnia, the burden or cost of illness, resource utilisations, and quality of life. There was a wide variation in the data within each of these categories because the studies differed in their definition of insomnia. The literature reviewed aided in the selection of the base case values and ranges for the incremental resource utilisations and outcomes.

From the international literature, insomnia prevalence was estimated at 5-35% [5]. This wide range in prevalence stems in most part from the many definitions of insomnia used in previous research. Thirty percent of individuals report symptoms of insomnia and 15-20% report insomnia symptoms with daytime impairment, whereas 5-10% meet criteria for a diagnosis of insomnia according to standardised diagnostic criteria [1,2]. New Zealand prevalence data align well with the international literature. Based on a national survey of insomnia symptoms [6,7], 25% of New Zealanders report having a sleep problem lasting longer than six months. From these data, we have estimated that 13% of New Zealanders are affected by at least one symptom of insomnia often/always, together with excessive daytime sleepiness [8]. Considerable disparity in estimated insomnia prevalence was observed between Māori (19.1%) and non-Māori (8.9%).

The burden of illness cost estimates for insomnia ranged from 0.2% to 0.5% of Gross Domestic Product (GDP), with a mean and median of 0.3% [9-13]. The Australian study [9] calculated that all sleep disorders represented 1.3% of GDP.

There were greater numbers of more recent studies that compared resource utilisations (direct and indirect) of individuals with insomnia with those of good sleepers. The differences in direct health costs between these two groups ranged from 5% to 200% (mean 57%, median 24%) [14-20]. Two high outliers [15,16] were eliminated resulting in a plausible range of 5-25%, with a mean of 18% and median of 21%. Insomniacs' absences from work (indirect costs) were higher by 15% to 142% (mean 86%, median 68%) compared with good sleepers [14,16,18,21].

The quality of life (QoL) studies in the international literature varied in the terminology they used to describe insomnia, some using descriptors that were not in accord with accepted diagnostic criteria. However, as most of the quality of life studies used the SF-36 on a scale of 0 to 100 points, the reduction in quality of life for the "physical functioning" and "mental health" domains/dimensions, or QoL scores, for insomniacs compared with good sleepers was able to be assessed [22-24]. Approximately 20% of all motor vehicle accidents are associated with driver sleepiness (independent of alcohol) [25]. Those reporting disrupted sleep were almost twice as likely (relative risk 1.89) to die in a work related accident [26] and 69% more likely to have a serious accident [27].

There were no reported studies of the proportion of insomniacs treated in New Zealand but findings from the United States suggest the majority of people (85%) who suffer from insomnia do not seek treatment [28]. A United Kingdom study [29] (sample size 85) investigated where insomniacs sought treatment and found that the providers most likely to have been consulted were; pharmacist (16.5%), general practitioner (41.2%), psychiatrist (3.5%), psychologist (7.1%), nurse (3.5%), counsellor (10.6%), herbalist (8.2%), acupuncturist (8.2%), and hypnotist (4.7%).

## Methods

This economic evaluation was a combination of cost effectiveness (CEA) and cost utility (CUA) analyses. The study used micro costing techniques, and was retrospective and prevalence-based. A societal analytic perspective was adopted and all costs were measured incrementally compared with the counterfactual (no intervention). As a time horizon of one year was used, discounting of costs and effects was not undertaken.

This study was informed by both the international literature and a series of key informant interviews [30] to canvas the range of treatment options offered in New Zealand and to estimate the proportion of people with insomnia who seek treatment. In order to ensure the interview data were representative of the range of insomnia diagnostic and treatment options available in New Zealand, informants were categorised as specialist physician (appropriately qualified physician working in specialty medical practice other than general practice), general practitioner (GP), psychologist, pharmacist, health practitioner (a medically-trained GP or other qualified health practitioner who has taken an interest, or undergone some training, in sleep) and alternative health practitioner (a treatment provider with any level of training in alternative medicine, practising insomnia treatment). An equal number of informants from each category were sought for interview.

Information was sought on the profile of patients (who had they previously consulted, number of new/referred patients, patient demographics), clinical practice (diagnosis, knowledge, treatments), patient outcomes (consultations, referrals, treatment effectiveness), and fees charged. Treatment effectiveness was self-rated by the interviewees and could not be independently verified.

The interviews were not sufficient to accurately describe insomnia patient treatment pathways and there is a paucity of data in the international literature. For the purposes of the model, findings from Stinson et al. [29] were used to estimate the percentage of patients approaching each provider type in the first instance. To correspond with the study of Stinson et al. (2006), patients approaching a nurse or counsellor were grouped in the category 'health practitioner', and patients approaching a herbalist, acupuncturist and hypnotist were grouped in the category 'alternative health practitioner'.

**Figure 1:**
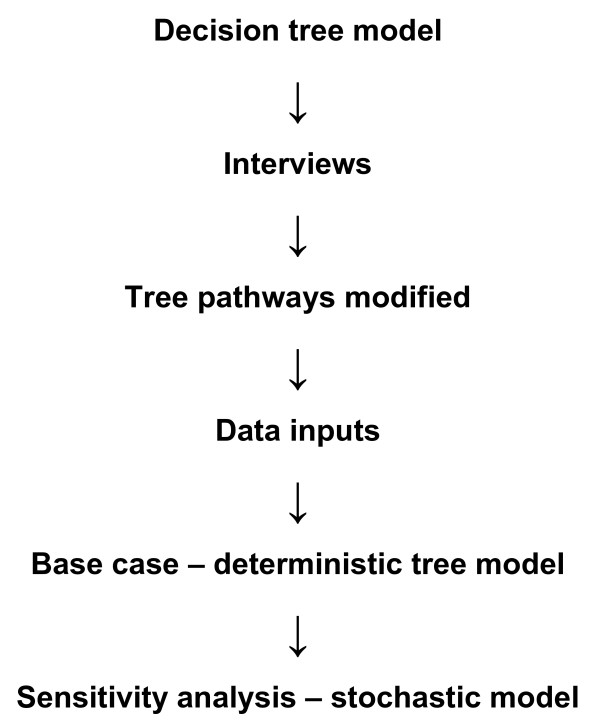
**Methodological steps**.

**Figure 2:**
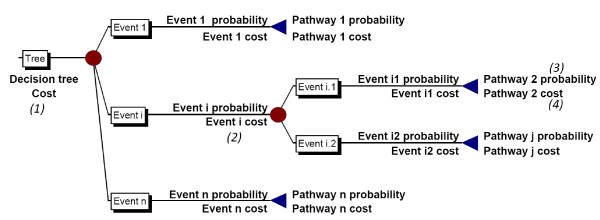
**Decision tree descriptions of calculations**. (1) Decision tree cost = the sum of all pathway costs (2) Cost of an event = the sum of (the unit costs of all resources utilised by the event multiplied by the volume of resources utilised) (3) Probability of a pathway = (pE_1 _× pE_2 _... xpE_I _... xpE_n_) where E_I _= event_I_, and n = the total number of events in the pathway (4) Cost of a pathway = the sum of the cost of all events in pathway (5) A decision tree enables a method of modelling, in chronological order, all possible events (6) Resources = consultations, medicines, and transport, E = event, p = probability (7) ● = Chance node which has a branch for each possible outcome or event. Each event ha s an associated probability and value. (8) ◀= End node which does not have any succeeding braches. Each end node returns a probability and a value for the associated pathway. Upper value = probability of reaching the end point of the pathway. Lower value = cost incurred in reaching the end point of the pathway.

A decision tree was developed to reflect treatment options for insomnia and modified when the findings of the key informant interviews were completed. The deterministic model developed was used to estimate base case values, and a stochastic version (with Monte Carlo simulation) was used to perform multivariate sensitivity analysis. Key methodological steps are shown in Figure 1. The decision tree represents a simplification of reality in that not every possible branch that a patient may follow has been included and the model was limited to one level of on-referral. The inputs to the model were prevalence, event probabilities, resource utilisations and unit costs. A schematic description of the calculations performed by the decision tree model is represented in Figure 2.

An individual with suspected insomnia may choose between two pathways; that is, they do not seek treatment or they seek treatment from a healthcare practitioner/provider. If they do not seek treatment, different outcomes may occur resulting in increased use of health resources, reduced productivity and reduced quality of life. The person with insomnia may have any or all of these outcomes in any combination. Based on the national prevalence data [6-8] the population at risk used for the model was 20-59 years (2.317 million) [31] and the prevalence of insomnia 13%. Event probabilities, costs and the referral pathways were determined from the literature [29] and interviews. While the international literature suggests that insomnia is associated with a range of other medical conditions, the cost of co-morbidities has not been included as the causal relationships between insomnia and comorbid conditions are not well understood.

Two of the insomnia practitioners who had participated in the interviews completed a EuroQol 5D (EQ-5D) questionnaire relating to their insomnia patients both before treatment and after practitioner-rated successful treatment. The EQ-5D was scored using the New Zealand-specific tariff (utility weights, tariff 2) [32]. The SF-36 scores for the two domains ("physical functioning" and "mental health") were converted from 0 - 100 to the scale 0 - 1 and then averaged. Scores from the literature taken from groups with the closest approximation to a standard clinical definition of insomnia (for example, 'severe" or "level II" insomnia) [19,22,23] and the EQ-5D clinician scores were combined into one dataset (range 0.078 to 0.373, mean 0.157). The dataset provided the base case (mean) and the high value, and 0 was assumed for the low value.

Direct medical provider costs and the indirect medical cost of transport to seek treatment were quantified but indirect costs (loss of productivity including travel time) and the non-health costs of accidents were not evaluated. It was assumed that the cost of any behavioural or psychological therapy, if given by any of the healthcare practitioners, was included in the fee for the initial visit. Unit resource cost estimates are described in table 1. The interviews provided data on medicines prescribed and this was supplemented with information from the Pharmaceutical Management Agency of New Zealand (PHARMAC) to cost the most prescribed medicine for insomnia, Zopiclone [33]. The interviews also supplied information on non-prescription products (over-the-counter preparations sold by Pharmacists) and the unit costs were taken from the website of Pharmacy Direct [34]. Blackmores Valerian Forte 2000 mg was used for the base case. Private motor vehicle costs incurred (travel for diagnosis and treatment) were calculated by multiplying the average cost per kilometre of $0.56 [35] by the average distance travelled for a round trip (29.83 km) to a GP or hospital clinic [36].

**Table 1:** Unit resource cost estimates in 2009 NZ dollars

Resource	Base case $	Year of data	Notes
**Direct medical**			
General Practitioner	48.89	2009	(1)
Specialist Physician initial	222.22	2009	(1)
Specialist Physician follow up	99.56	2009	(1)
Psychologist	88.89	2009	(1)
Health Practitioner	120.00	2009	(1)
Alternative Health Practitioner	75.56	2008	(2)
Prescription medicine	6.42	2009	(3)
Non prescription medicine	16.00	2009	(4)
Increase in cost per capita for those with insomnia versus non-insomniacs	627.52	2008	(5)
**Direct non-medical**			
Transport for treatment (round trip)	16.71	2009	(6)

Notes:

(1) Registered health care providers [40]. General Practitioner, medical practitioner band 1. Specialist Physician, medical practitioner band 2; high case (interviews). The medical fees do not include any government patient subsidy as this varies between providers and patients.

(2) Alternative Health Practitioner from Interviews

(3) Prescription medicine, Zopiclone, base case 7.5 mg @ 30 days plus dispensing fee, low case = base case × 0.5 plus dispensing fee, high case = two prescriptions plus 2 dispensing fees (Interviews) and dispensing fee [39], prices [33]

(4) Non prescription medicine (Interviews) and [34], low case = base case less 25%, high case = base case × 2

(5) See Table 3

(6) Transport for treatment: Cost per km × km travelled for round trip = $0.63/9 × 8 × 29.83 km = $16.71. [Cost per km $0.63/9 × 8: 1500-2000 cc petrol: [35]. Time to hospital (17.9 minutes): [36]. Distance for round trip (km): 17.9 minutes @ 50 km/hour × 2 = 29.83 km.]

Ranges: if not specifically stated ranges = base case plus or minus 25%

All costs have had GST of 12.5% deducted and calculations are based on unrounded data.

**Figure 3:**
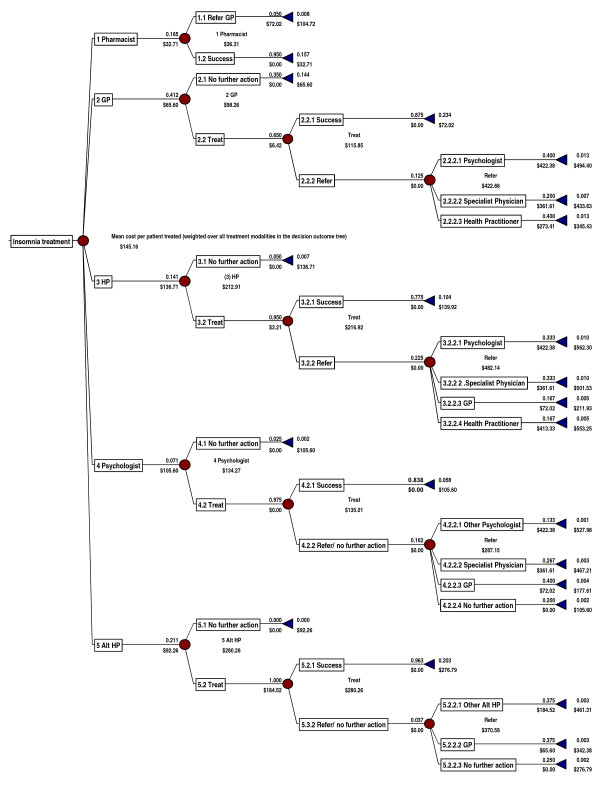
**Insomnia treatment model**.

The event probabilities are summarised in Figure 3. At each node choices are made, events take place and resource utilisations are changed. If, for example, a person who has insomnia consults their Pharmacist they may purchase an over-the-counter (OTC) medication and incur transport costs. (See table 2) The average increase in health resource utilisations for those with insomnia versus non-insomniacs were derived from the literature (table 3).

**Table 2:** Resource utilisations by event

Events	(a)	(b)	(c)	(d)	(e)	(f)	(g)	(h)	(i)
Do not seek treatment									
Seek treatment									
1 Pharmacist								1.0	1.0
1.1 Refer General Practitioner	1.0						1.0		1.0
1.2 Success									
2 General Practitioner	1.0								1.0
2.1 No further action									
2.2 Treat							1.0		
2.2.1 Success									
2.2.2 Refer									
2.2.2.1 Psychologist				4.0					4.0
2.2.2.2 Specialist Physician		1.0	1.0				1.0		2.0
2.2.2.3 Health Practitioner					2.0				2.0
3 Health Practitioner					1.0				1.0
3.1 No further action									
3.2 Treat							0.5		
3.2.1 Success									
3.2.2 Refer									
3.2.2.1 Psychologist				4.0					4.0
3.2.2.2 Specialist Physician		1.0	1.0				1.0		2.0
3.2.2.3 General Practitioner	1.0						1.0		1.0
3.2.2.4 Other Health Practitioner					3.0		0.5		3.0
4 Psychologist				1.0					1.0
4.1 No further action									
4.2 Treat									
4.2.1 Success									
4.2.2 Refer/no further action									
4.2.2.1 Other Psychologist				4.0					4.0
4.2.2.2 Specialist Physician		1.0	1.0				1.0		2.0
4.2.2.3 General Practitioner	1.0						1.0		1.0
4.2.2.4 No further action									
5 Alternative Health Practitioner						1.0			1.0
5.1 No further action									
5.2 Treat						2.0			2.0
5.2.2 Success									
5.2.3 Refer/no further action									
5.2.3.1 Other Alternative Health Practitioner						2.0			2.0
5.2.3.2 General Practitioner	1.0								1.0
5.2.3.3 No further action									

Notes:

Events: (a) General Practitioner consultation, (b) Specialist Physician Initial consultation, (c) Specialist Physician follow-up consultation, (d) Psychologist consultation, (e) Health Practitioner consultation, (f) Alternative Health Practitioner consultation, (g) Prescription medicine, (h) Non prescription medicine, (i) Transport for treatment (round trip)

Base case values were derived from the current study. Monte Carlo simulation runs used base case, and ranges base case plus or minus 25%.

**Table 3:** Health care cost of those with insomnia versus non-insomniacs

Item	Base Case	Year of data	Notes
Per capita health care resource cost ($) of all ages New Zealand population (TP$)	3,568	2008	(1)
Personal medical services ($M)	15,313	2008	(2)
Population all ages (M)	4.292	2008	(3)
Proportion of New Zealand population suffering from insomnia (Ip)	0.13		(4)
% Increase in cost per capita of those with insomnia versus non-insomniacs	18.0%		(5)
Ratio of health resource cost of those with insomnia to non-insomniacs (R)	1.18		(5)
Mean health care resource cost ($) of non-insomniacs (Y)	3,486		(6)
Mean health care resource cost ($) of those with insomnia (X)	4,114		(6)
Increase in cost per capita those with insomnia versus non-insomniacs	628		(6)

Notes:

Data sources

(1) = (2) ÷ (3)

(2) Personal medical services: excludes expenditure on prevention and public health, administration and insurance premiums [41].

(3) Population: Total resident population[31]

(4) [8]

(5) [14,17-20]

(6) Derivation of "Y" and "X" from "Ip" "R" and TP$

Unknown

X = Mean health care resource cost ($) of those with insomnia

Y = Mean health care resource cost ($) of non-insomniacs

Known (Statements S1, S2, S3)

(S1): Ip = Proportion of New Zealand population suffering from insomnia, [base case 0.13]

(S2): R = Ratio of health resource cost of those with insomnia to others, [base case 1.18]

(S3): TP$ = Mean health care resource cost of total all ages New Zealand population, [base case $3,568]

Solution

(S1) and (S3) may be used to derive equation (E1): TP$ = Ip × X + [(1 - Ip) × Y] (S2) may be written as equation (E2): × = R × Y

Substitute (E2) into (E1)

TP$ = [Ip × R × Y] + [(1 - Ip) × Y]

TP$ = Y × [(Ip × R) +1 - Ip)]

Solve for Y

Y = TP$/[(Ip × R) + 1 - Ip]

Using base case values as an example

Y = $3,568/[(0.13 × 1.18)+1-0.13] = $3,486

X = ($3,486 × 1.18) = $4,114

All calculations are based on unrounded data.

Sensitivity analysis (rather than statistical methods) was used to investigate uncertainty in the model inputs (unit costs, resource utilisations, QALYs, and prevalence). A stochastic version of the insomnia costing model, using Monte Carlo sampling from triangular distributions, was used for multivariate sensitivity analysis [37]. Triangular distributions were used because there was insufficient information from which to define specific distributions (for example, normal or Pareto distributions). Unless otherwise stated, all estimates subject to uncertainty were varied 25% up and down from the base case to provide high and low limits. Ten thousand iterations of the model were run. The Monte Carlo simulations used Palisade's Decision Tools Suite software.

All unit costs were valued in 2009 NZ dollars (or the latest available data) and were exclusive of GST (goods and services tax, a transfer payment from one sector of society to another). New Zealand dollar conversions; mid rates end Dec 2009 NZD1 = AUD0.7929, €0.4901, USD0.7162 [38].

## Results

The interviews revealed little awareness of international best practice standards for insomnia treatment. Alternative health practitioners, pharmacists and GPs (when compared with specialist physicians, health practitioners) and had poorer knowledge of the types of insomnia and sleep terminology, were less likely to use any structured diagnostic tools and offered the most limited range of treatment options. The effectiveness of treatment provided was formally assessed by 57%. The interviewees suggested that patients had frequently consulted multiple practitioners. It was considered that there was an unmet need for insomnia treatment and a lack of accurate information on treatment options and providers.

The decision tree (Figure 3) is the final version developed and used for modelling treatment pathways and costs. The pathways and events depicted are the dominant and most relevant for which local data existed. Treatment cost over all treatments (the tree cost) averaged $145 per patient. The mean treatment cost for each branch or mode of treatment designated by the health practitioner first consulted was as follows; pharmacist $36, GP $98, psychologist $134, health practitioner $213, and alternative health practitioner $280. The total direct costs for each treatment outcome or pathway that ended in a termination node ran from a low of $33 (cost of an OTC product and travel, pharmacist pathway) to a high of $562 (psychologist accessed through a health practitioner pathway). The direct costs of treatment by a specialist physician depended upon the referral pathway taken and ranged from $434 (accessed through a GP) to $502 (when accessed through a health practitioner).

The total net benefit of treating a person with insomnia was $482 (the total base case cost of $145 less costs avoided of $628). When these results were applied to the total at-risk population in New Zealand treatment costs incurred were $6.6 million, costs avoided $28.4 million and net benefits were $21.8 million. The incremental net direct benefit per QALY gained when insomnia was successfully treated was $3,072 (table 4). When multivariate sensitivity analysis was undertaken on the net benefits of treatment, it was found that for 90% of the Monte Carlo 10,000 simulations the net benefit of treatment per person fell between $41 and $679, and for New Zealand as a whole between $2 million to $33 million. The net benefit per QALY gained ranged between $240 and $8,102.

**Table 4:** Economic evaluation of insomnia treatment versus no treatment

	Per person treated	NZ total million
At risk population	(1)	2.317
Prevalence of insomnia	(2)	13%
Proportion seeking treatment	(2)	15.0%
Number seeking treatment (M)	(3)	0.045
Costs incurred ($)	145	6.6
Costs avoided ($)	628	28.4
Net benefit ($)	482	21.8
QALYs gained (#)	0.157	0.007
Net benefit per QALY gained ($)	3,072	21.8

Notes:

(1) December 2008 [31]

(2) [8]

Ranges (1) and (2) The high values are the base case plus 25% and the low values the base case minus 25%.

(3) = (1) × (2) × (3)

Calculations are based on unrounded data.

## Discussion

We now have a greater understanding of the treatment of insomnia in New Zealand in terms of the types of diagnostic and treatment options being used. Both the information on the impact on health resource utilisation and improved quality of life (if insomnia is successfully treated) should assist in identifying cost-effective treatments and policies. The model developed may be used to investigate population subgroups and evaluate different treatment options.

The cost of each treatment path varied, depending not only on the fees charged and the number of consultations per course of treatment but also upon the number of encounters with different healthcare practitioners/treatment providers (referrals). The study assumed a successful treatment outcome (no further additional impact on health resource utilisations) at each termination node. As individuals with insomnia are more likely to consult their Pharmacist or GP in the first instance, it is important that both these practitioners have clear guidelines and protocols to identify potential insomnia and where appropriate, on-refer a patient to a trained treatment provider.

There are no publicly funded treatment options for insomnia in New Zealand. Market failure caused by insufficient patient information is indicated in that the interviews found that it was not uncommon for patients with insomnia to have independently consulted several practitioners. It was considered that those with insomnia lacked sufficient accurate and unbiased information from which they were able to make an informed decision.

Sensitivity analysis demonstrated that the results were robust with respect to changes in key assumptions and determinants of cost and effects (over 90% of all iterations were both more effective and less costly). By way of comparison, the cumulative average cost-effectiveness threshold of PHARMAC funding decisions for new medicines made between 1999 and 2005 was $6,865 [39].

The study is the first in New Zealand to attempt to ascertain the treatment pathways that a person with insomnia may follow. It also sought to understand the treatment provided (based on interviews of healthcare practitioners) and to quantify the costs of insomnia and its impact on quality of life.

### Limitations

Individuals with insomnia access health care services more often than others, and insomnia is associated with a range of other medical conditions. However, the causal relationship between insomnia and these comorbid conditions is not well understood. Thus, the costing model in this study did not specifically account for conditions that may be caused by insomnia but instead evaluated the impact on total health resource utilisation, using information from international studies. Costs of non-prescription medicines from health food stores and supermarkets or from web-based vendors were not considered.

Individuals with insomnia may be at increased risk for decreased performance and accident or injury. This is best described in relation to motor vehicle accidents; those with insomnia have a higher motor vehicle accident rate than controls [27]. However, the relative risk of decreased performance in those with insomnia is not well understood. This study has taken a conservative approach. For example, a person with insomnia has increased non-health costs of having an accident, injuring others and/or damaging property but this was not considered. In addition, the analyses did not quantify externalities that impact on others in the community such as the effect on productivity and quality of life of having an insomniac within a family setting. Thus, we did not calculate the burden of illness as a percentage of GDP.

As healthcare practitioners were interviewed (and not patients), it is their judgements on pathways, and patient outcomes that have been used to define the model. Thus, the true success of treatment by these providers remains unknown. Costs incurred by those 60 years and older were not included as the at risk population was limited by the available insomnia prevalence estimates for New Zealanders aged 20-59 years.

### Recommendations

A larger nationwide survey of those offering insomnia treatments would provide a greater understanding of diagnostic, treatment and referral practices in New Zealand, and would give a more comprehensive sample on which to base QALY estimates. A large survey of individuals in New Zealand who identify as having insomnia would provide a means to more accurately identify the difference in healthcare resource utilisations and productivity between those people with untreated insomnia, treated insomnia and those without insomnia and to determine patients' evaluation of the effectiveness of the various treatments and treatment pathways followed. The existing model, augmented by additional national survey data, could be modified and used to evaluate the cost of alternative funding policies and treatment options.

## Conclusions

The interviews highlighted the unsystematic approach to insomnia treatment in New Zealand. It is concerning that there is insufficient publicly available information from which a consumer is able to make an informed decision on treatment provider options and provider competence. A standardised approach to insomnia treatment requires a multi-disciplinary team of treatment providers who have sufficient knowledge to diagnose insomnia, implement treatment and measure treatment efficacy. This would reduce the direct and indirect costs of insomnia and improve quality of life.

A number of study limitations resulted in a conservative estimate of the costs of insomnia treatment in New Zealand. Despite this conservative approach, this study confirms that successful treatment of insomnia is highly cost effective.

## Competing interests

The authors declare that they have no competing interests.

## Authors' contributions

All authors contributed equally to the study and all have read and approved the final manuscript.
